# An Efficient and Provable Secure Revocable Identity-Based Encryption Scheme

**DOI:** 10.1371/journal.pone.0106925

**Published:** 2014-09-19

**Authors:** Changji Wang, Yuan Li, Xiaonan Xia, Kangjia Zheng

**Affiliations:** 1 School of Information Science and Technology, Sun Yat-sen University, Guangzhou, China; 2 Guangdong Province Information Security Key Laboratory, Sun Yat-sen University, Guangzhou, China; Tianjin University of Technology, China

## Abstract

Revocation functionality is necessary and crucial to identity-based cryptosystems. Revocable identity-based encryption (RIBE) has attracted a lot of attention in recent years, many RIBE schemes have been proposed in the literature but shown to be either insecure or inefficient. In this paper, we propose a new scalable RIBE scheme with decryption key exposure resilience by combining Lewko and Waters’ identity-based encryption scheme and complete subtree method, and prove our RIBE scheme to be semantically secure using dual system encryption methodology. Compared to existing scalable and semantically secure RIBE schemes, our proposed RIBE scheme is more efficient in term of ciphertext size, public parameters size and decryption cost at price of a little looser security reduction. To the best of our knowledge, this is the first construction of scalable and semantically secure RIBE scheme with constant size public system parameters.

## Introduction

Shamir [1] first introduced the concept of identity-based public key cryptography (ID-PKC) where a public key can be an arbitrary string such as an email address or a telephone number, while the corresponding private key can only be generated by a private key generator (PKG) who has the knowledge of the master secret. The first secure and practical identity-based encryption (IBE) scheme was proposed by Boneh and Franklin [2] from bilinear pairings, which is proved to be semantically secure against adaptive chosen ciphertext attack (IND-ID-CCA) under the Decisional Bilinear Diffie-Hellman (DBDH) assumption in the random oracle model.

Boneh and Franklin's work spurred a great deal of research on IBE. One important research direction is to construct provably secure IBE schemes in the standard model, because random oracle model only provides heuristic security [3]. Canetti, Halevi, and Katz [4] defined a weaker security notion for IBE, known as selective-ID model, in which the adversary commits ahead of time to the identity that it intends to attack. Boneh and Boyen [5] proposed two efficient IBE schemes that are secure in the selective-ID model without random oracle. The first IBE construction (BB1-IBE) is based on the DBDH assumption, while the second IBE construction (BB2-IBE) is based on a non-standard Decision Bilinear Diffie-Hellman Inversion (DBDHI) assumption. Waters [6] improved BB1-IBE scheme and proposed an efficient IBE scheme which is proved to be semantically secure without random oracles under the DBDH assumption in adaptive-ID model. Gentry [7] presented an IBE scheme with short public parameters which is proved to be semantically secure without random oracles under a non-static assumption in adaptive-ID model. Waters [8] introduced a new technique called dual system encryption and proposed an IBE scheme that is proved to be semantically secure without random oracle under standard (static) assumption in adaptive-ID model. Recently, Lewko and Waters [9] gave a new dual system encryption realization of IBE from composite order bilinear groups, which is proved to be semantically secure without random oracle under the subgroup decision assumption in adaptive-ID model.

Another important research direction is to construct IBE schemes with efficient revocation. Suppose that Alice has left the organization or her private key is compromised or stolen by an adversary in some scenarios [10]. On the one hand, Alice will be withdrawn from the right of accessing the information with respect to her public key. On the other hand, Alice's private key will be revoked to prevent the adversary with her compromised private key to access confidential data encrypted under her public key. Thus, revocation functionality is necessary and crucial to public-key cryptosystems. In the public key infrastructure setting, numerous solutions have been proposed, such as periodic publication mechanisms (e.g. certificate revocation list) and online query mechanisms (e.g. online certificate status protocol). In the ID-PKC setting, however, key revocation is non-trivial. This is because a user's identity is itself a public key, thus one can not simply change her public key, as this changes her identity as well. An ideal revocation method for IBE is that a sender can generates a ciphertext as the same as that of IBE without worrying about the revocation of a receiver and only the receiver needs to check the revocation of his private key to decrypt the ciphertext.

Revocable IBE (RIBE) has attracted a lot of attention in recent years, many RIBE schemes have been proposed [2], [11]–[15]. Boneh and Franklin [2] proposed a trivial method to achieve revocation functionality for IBE (BF-RIBE for short) by representing an identity as 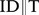 where 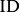 is the real identity and 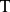 is a current time. Since new decryption keys are needed to be issued by the PKG for each time period, this introduces huge overheads for PKG that are linearly increased in the number of users and a secure channel is needed between PKG and users to transmit updated private key. Thus, BF-RIBE is not scalable.

**Figure 1:**
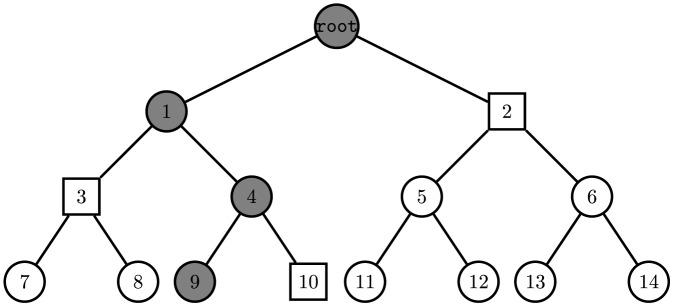
Example of KUNode Algorithm. Assume that the user associated with node 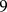 is revoked. As figure illustrated, user assigned to leaf node 7 has subkeys of node 7, 3, 1 and root. In time period 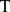, only user assigned to leaf node 9 is revoked, the square nodes are update nodes set outputted by the KUNode algorithm, it's obvious that this set does not contain any node on the path from node 9 to root node.

Boldyreva et al. [11] proposed the first scalable RIBE scheme (BGK-RIBE for short) by combining Sahai and Waters' fuzzy IBE scheme [16] and Naor et al.'s complete subtree method [17], where the PKG's overhead increases logarithmically (instead of linearly) in the number of users. The idea of BGK-RIBE scheme consists in assigning users to the leaves of a complete binary tree. Each user is provided by PKG with a set of private keys 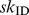 corresponding to his/her identity 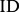 for each node on the path from his/her associated leaf to the root of the tree via a secure channel as in IBE scheme. PKG broadcasts key updates 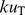 in each time period 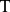 for a set 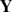 of nodes that contains no ancestors of revoked users and exactly one ancestor of any non-revoked one (as illustrated in Figure 1 where the nodes of 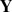 are the squares). Then, a user assigned to leaf 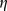 is able to form an effective decryption key 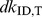 for period 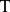 if the set 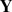 contains a node on the path from the root to 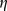. By doing so, every update of the revocation list 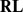 only requires PKG to perform logarithmic work in the overall number of users and no secure channel is required between PKG and users. The size of users' private keys also logarithmically depends on the maximal number of users.

Another idea of BGK-RIBE scheme consists in applying fuzzy IBE primitive. In fuzzy IBE systems, identities are regarded as sets of descriptive attributes instead of a single identity string in IBE systems, and a user with private key for the attribute set 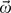 is able to decrypt a ciphertext encrypted for an attribute set 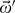 if and only if 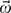 and 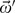 have an overlap of at least 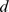 attributes. The BGK-RIBE scheme uses a special kind of fuzzy IBE where ciphertexts are encrypted using the receiver's identity and the period number as “attributes”. The decryption key of the receiver has to match both attributes to decrypt the ciphertext. For each node on the path from the root to its assigned leaf, the user is given a key attribute that is generated using a new polynomial with degree 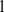 for which the constant term is always the master secret. The same polynomials are used, for each node, to generate key updates. To compute a decryption key for period 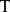, each user thus needs to combine two key attributes associated with the same node of the tree. Since there is no adaptive-ID secure fuzzy IBE scheme in the literature, BGK-RIBE scheme [11] is only proved to be secure in selective-ID model.

Later, Libert and Vergnaud [12] proposed the first adaptive-ID secure scalable RIBE scheme (LV-RIBE for short) based on same idea as BGK-RIBE scheme, but, instead of using fuzzy IBE scheme, they applied the idea of two-level hierarchial IBE scheme (HIBE for short). They use adaptive-ID secure Libert and Vergnaud's black-box accountable authority IBE scheme [18] in the first level to handle user's long term private keys (associated with identities), and use selective-ID secure Boneh and Boyen's BB1-IBE scheme [5] in the second level to handle decryption keys (associate with time periods). Seo and Emura [13] refined the security model of RIBE by considering the decryption key exposure attacks, and proposed a scalable RIBE scheme (SE-RIBE for short) with decryption key exposure resistance based on same idea as LV-RIBE scheme. Seo and Emura use adaptive-ID secure Waters IBE scheme [6] in the first level to handle user's long term private keys, and use selective-ID secure BB1-IBE scheme [5] in the second level to handle decryption keys. Recently, Park et al. [14] proposed a scalable RIBE scheme with shorter private key and update key by using multilinear maps, but the size of the public parameters is dependent to the number of users. Lee et al. [15] presented a new technique for RIBE that uses the subset difference method instead of using the complete subtree method to improve the size of update keys.

Existing adaptive-ID secure scalable RIBE constructions are built on combining two-level HIBE schemes and complete subtree method, and proved security with partition strategy in which the space of identities is partitioned into the set of identities for which a valid secret key can be simulated and those for which a valid challenge ciphertext can be simulated.

In this paper, we propose an efficient adaptive-ID secure scalable RIBE scheme by combinineg two-level Lewko and Waters HIBE scheme [9] and complete subtree method. To prove security for our RIBE scheme in adaptive-ID model, we adopt Waters dual system encryption methodology [8]. However, we can not use dual system encryption methodology directly to prove the security of RIBE schemes. This is because an adversary in RIBE schemes can issue private key query for the challenge identity 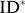 as long as 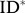 has been revoked before the challenge time 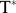, while an adversary in IBE schemes can not issue private key query for the challenge identity 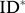. Furthermore, as stated in Seo and Emura [13], an adversary in a scalable RIBE scheme with decryption key exposure resistance may obtain not a private key 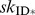 but a decryption key 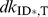, and 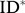 can still be alive in the system in the challenge time period 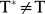.

To make dual system encryption methodology work properly, we need to make sure that all decryption keys, including those generated by the adversary, are semi-functional in the last step. It is not a trivial job to accomplish this transformation directly. To circumvent this issue, our approach is to design semi-functional private key and semi-functional update key, and generate a semi-functional decryption key from a semi-functional private key or a semi-functional update key.

During registration, PKG assigns a user with identity 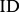 to a leaf node 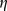 of a complete binary tree, and issues the private key 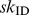 for identity 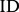 which is composed by a set of subkeys 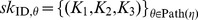, wherein each subkey is associated with a node on 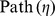. At time period 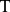, PKG broadcasts the update key 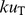 which is composed by a set of subkeys 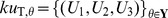, wherein each subkey is associated with a node in 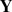. An intuitive way to make all decryption keys be semi-functional in the last step is to transform all subkeys of all private keys or all subkeys of update keys from normal form into semi-functional form. However, similar to the security proof in Lewko and Waters' IBE scheme [9], the adversary cannot issue private key query for identities which are equal to the challenge identity 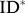 modulo 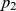, and cannot issue update key query for time periods which are equal to the challenge time 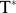 modulo 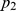, namely all subkeys of these private keys and all subkeys of these update keys can not be transformed. On the one hand, if we transform either all subkeys of the corresponding private key 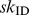 satisfying 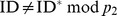 or all subkeys of the corresponding update key 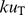 satisfying 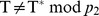 from normal form into semi-functional form independently, the resulting decryption keys 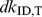 may not be semi-functional. On the other hand, if we transform all subkeys of the corresponding update key 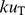 from normal form into semi-functional form, this will result in security degradation 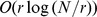 when 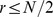, and security degradation about 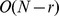 when 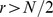, where 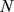 is the number of users and 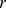 is the number of revoked users.

To solve the problem of security degradation, we take advantage of the special structure of complete subtree method. We do not need to transform all subkeys of 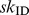 that satisfy 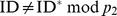 and all subkeys of 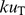 that satisfy 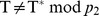 from normal form into semi-functional form, we just need to transform subkeys of above 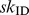 that satisfy 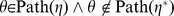, and subkeys of above 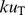 that satisfy and 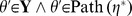 from norm form into semi-functional form, where 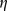 and 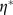 are leaf nodes of binary tree that assigned to 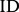 and 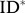, respectively. Thus, security degradation is reduced to 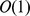 per transformation of a update key.

Compared to existing adaptive-ID secure scalable RIBE schemes, our RIBE scheme is more efficient in term of ciphertext size, public parameters size and decryption cost at price of a little looser security reduction. To the best of our knowledge, this is the first construction of scalable semantically secure RIBE scheme with constant size public system parameters. Table 1 shows a comparison between our RIBE scheme and existing RIBE schemes.

**Table 1:** Comparison among RIBE schemes.

RIBE schemes	Security model	Complexity assumption	Scalability	DKE resistance	CT size	Dec. cost	Mpk size
BF-RIBE [2]	Adaptive RO	DBDH	No	Yes	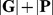	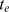	3 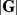
BGK-RIBE [11]	Selective RO	DBDH	Yes	No	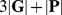	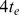	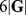
LV-RIBE [12]	Adaptive Standard	DBDH	Yes	No	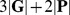	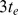	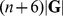
SE-RIBE [13]	Adaptive Standard	DBDH	Yes	Yes	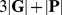	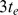	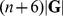
Our RIBE	Adaptive Standard	SGD	Yes	Yes	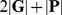	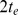	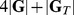

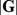
 and 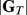 are the sizes of groups 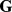 and 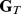, respectively. 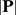 is the size of plaintext space, and 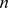 is the size of identity space. 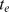 is the cost for performing a bilinear pairing 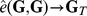. Selective (Adaptive, respectively) is a selective-identity security model (adaptive-identity security model, respectively). RO (Standard, respectively) is a random oracle model (standard model, respectively). DBDH is Decisional Bilinear Diffie-Hellman assumption, and SGD is Subgroup Decision assumption. DKE is decryption key exposure.

The rest of the paper is organized as follows. In Section 2, we introduce some preliminary works necessary for our constructions, such as bilinear group generator and complexity assumptions. In Section 3, we give formal syntax and security definitions of RIBE. In Section 4, we describe our RIBE construction. In Section 5, we prove our RIBE construction are IND-RID-CPA secure. Finally, we conclude the paper in Section 6.

## Preliminaries

### Bilinear group generator and complexity assumptions

#### Definition 1

(Bilinear Group Generator) *A bilinear group generator* 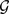 *is an algorithm that takes as input a security parameter* 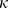 *and outputs a bilinear group* 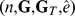 *, where* 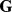 *and* 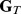 *are cyclic groups of order* 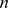 *, and* 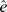 *:* 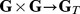 *is a bilinear map with the following properties:*

- *Bilinearity: For all* 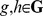 *and* 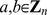 *, we have* 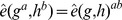 *.*
- *Non-degeneracy: There is an element* 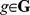 *such that* 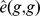 *has order* 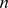 *in* 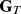 *.*
- *Computability: There is an efficient algorithm to compute* 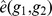 *for all* 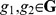 *.*

Denote 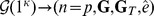 a prime order bilinear groups generator, where 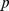 is a prime. We call 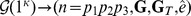 a composite order bilinear groups generator, where 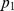, 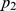 and 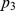 are distinct primes. The subgroups of order 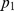, 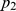 and 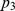 in 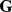 are denoted by 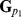, 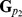 and 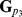, respectively. Note that when 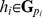 and 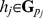 for 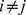, we have 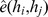 is the identity element in 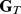.

#### Definition 2

(Decision Bilinear Diffie-Hellman Assumption) *Given a prime order bilinear group* 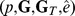 *generated by* 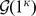 *, we define the following two distributions:*



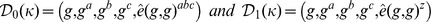

*where* 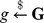 *and* 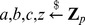 *. The DBDH problem in the prime order bilinear group* 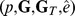 *is to decide a bit* 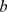 *from given* 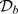 *, where* 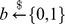 *. The advantage of an algorithm* 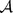 *in solving the DBDH problem in the prime order bilinear group* 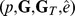 *is defined by*



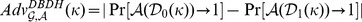



*We say that the DBDH assumption holds in the prime order bilinear group* 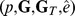 *if no probabilistic polynomial time (PPT) algorithm has a non-negligible advantage in solving the DBDH problem in the prime order bilinear group* 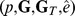 *.*

#### Assumption 1

(Subgroup decision problem for 3 primes) *Given a composite order bilinear group generator* 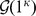 *, we define the following two distributions:*



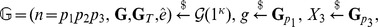


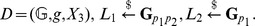



We define the advantage of an algorithm 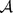 in breaking the subgroup decision assumption 1 to be:



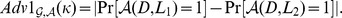



We note that 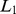 can be written (uniquely) as the product of an element of 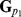 and an element of 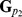. We refer to these elements as the “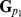 part of 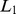
” and the “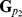 part of 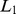
” respectively.

#### Definition 3

*We say that* 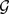 *satisfies the subgroup decision Assumption 1 if* 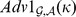 *is a negligible function of* 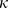 *for any polynomial time algorithm* 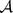 *.*

#### Assumption 2

(Subgroup decision problem for 3 primes) *Given a composite order bilinear group generator* 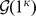 *, we define the following two distributions:*



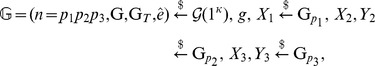


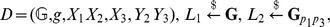



*We define the advantage of an algorithm* 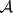 *in breaking the subgroup decision assumption 2 to be:*



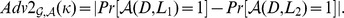



#### Definition 4

*We say that* 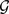 *satisfies the subgroup decision Assumption 2 if* 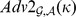 *is a negligible function of* 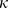 *for any polynomial time algorithm* 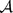 *.*

#### Assumption 3

(Subgroup decision problem for 3 primes) *Given a composite order bilinear group generator* 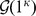 *, we define the following two distributions:*



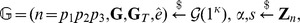


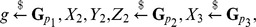


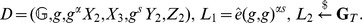



*We define the advantage of an algorithm* 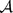 *in breaking the subgroup decision assumption 3 to be:*



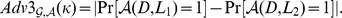



#### Definition 5

*We say that* 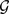 *satisfies the subgroup decision Assumption 3 if* 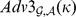 *is a negligible function of* 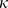 *for any polynomial time algorithm* 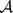 *.*

### KUNode Algorithm

The KUNode algorithm was proposed by Boldyreva et al. [11] to achieve efficient revocation for IBE schemes. In the description hereafter, we employ similar notations as in [11]. Denote the root node of the tree 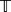 by root. If 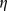 is a leaf node, we denote the set of nodes on the path from 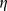 to root by 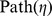. If 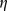 is a non-leaf node, we denote the left and right child of 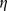 by 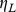 and 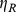, respectively.

At each time period, KUNode algorithm determines the smallest subset 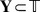 of nodes that contains an ancestor of all leaves corresponding to non-revoked users. This minimal set precisely contains nodes for which key updates have to be publicized in such a way that only non-revoked users will be able to generate the appropriate decryption key for the matching period. To identify the set 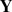, KUNode algorithm takes as input a binary tree 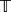, revocation list 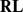 and a period number 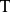. If a user (assigned to 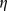) is revoked on time 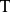, then 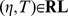. KUNode algorithm first marks all ancestors of users that were revoked by time 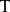 as revoked nodes. Then, it inserts in 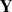 the non-revoked children of revoked nodes. The description of 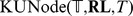 is given in Table 2 Algorithm 2.

**Table 2:** Algorithm 2: KUNode Algorithm 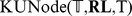.

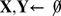 .
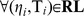
**if** 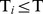 **then**
Add 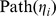 to 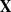
**end if**
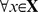
**if ** 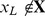 **then**
Add 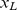 to 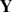
**end if**
**if ** 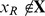 **then**
Add 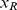 to 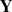
**end if**
**if** 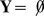 **then**
Add root to 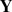
**end if**
Return 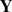

The example illustrated in Figure 1 can be used to help the reader understand the 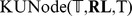 algorithm. Assume that a user associated with node 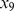 is revoked, then 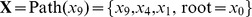 and 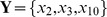. Intuitively, all users, except the user associated with noed 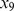, have a node 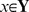 that is contained in the set of nodes on the path from their assigned node to root, whereas 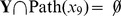.

When a user joins the system, PKG assigns a leaf node 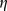 of a complete binary tree to the user, and issues a set of keys, wherein each key is associated with a node on 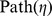. At time period 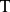, PKG broadcasts key updates for a set 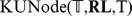. Then, only non-revoked users have at least one key corresponding to a node in 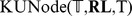 and are able to generate decryption keys on time 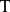.

### Dual System Encryption

Dual system encryption is a proof methodology first introduced by Waters [8], which opens up a new way to prove adaptive security under simple assumptions for IBE and related encryption systems.

In a dual system encryption system, both ciphertexts and private keys can take on one of two indistinguishable forms [9]. A private key or ciphertext is normal if they are generated from the system's key generation or encryption algorithm. Semi-functional ciphertexts and private keys are not used in the real system, they are only used in the security proof. A normal private key can decrypt normal or semi-functional ciphertexts, and a normal ciphertext can be decrypted by normal or semi-functional private keys. However, decryption will fail with high probability if one attempts to decrypt a semi-functional ciphertext with a semi-functional private key.

Unlike previous proof technique called partitioning strategy which partitions the identity space into two parts, dual system encryption defines a sequence of games and proves their indistinguishability with the real game. The first game is the real security game in which the challenge ciphertext and private keys are normal. In the next game, the ciphertext is switched from normal to semi-functional, while all the private keys are normal. For an adversary that makes 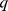 private key requests, games 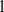 through 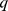 follow. In game 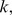 the first 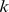 private keys are semi-functional while the remaining private keys are normal. In game 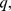 all the private keys and the challenge ciphertext given to the adversary are semi-functional. Hence none of the given private keys are useful for decrypting the challenge ciphertext. At this point, At this point proving security becomes relatively easy since the reduction algorithm does not need to present any normal private keys to the adversary and all semi-functional private keys are useless for decrypting a semi-functional ciphertext.

## Syntax and Security Definitions of RIBE

In this section, we recall the syntax and security model of RIBE as defined in [13]. Unlike the syntax definition in [13], we define the decryption key generation algorithm as probabilistic rather than deterministic. A RIBE scheme can be defined by the following seven polynomial-time algorithms:

**Setup** The stateful setup algorithm is run by the PKG, which takes a security parameter 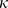 and a maximal number of users 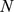 as input, it outputs the public parameter 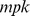, the master secret key 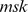, the initial revocation list 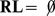, and a state 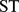. We assume that the message space 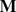 and the identity space 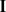, the time space 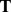, and the ciphertext space 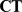 are contained in 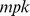.

**Extract** The stateful private key extract algorithm is run by the PKG, which takes 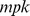, 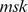, an identity 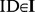, a state 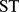 as input, it outputs a secret key 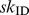 associated with 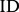 and an updated state 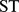.

**KeyUpdate** The key update generation algorithm is run by the PKG, which takes 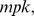 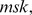 the key update time 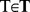, the current revocation list 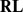, and 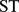 as input, it outputs the key update 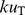.

**DKeyGen** The probabilistic decryption key generation algorithm is run by a user, which takes 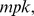 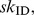 and 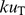 as input, it outputs a decryption key 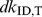 to be used during period 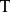 or a special symbol 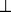 indicating that 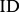 was revoked.

**Encrypt** The probabilistic encryption algorithm is run by a sender, which takes 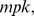 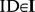, 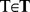, and a message 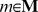 as input, it outputs a ciphertext 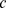.

**Decrypt** The deterministic decryption algorithm is run by the receiver, which takes 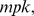 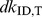, and 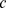 as input, it outputs 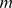 or 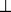 if 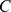 is an invalid ciphertext.

**Revoke** The stateful revocation algorithm is run by the PKG, which takes an identity to be revoked 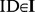, a revocation time 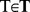, the current revocation list 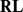, and a state 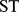 as input, it outputs an updated 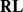 by adding 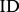 as a revoked user at time 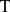.

We have a basic consistency requirement that for any 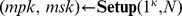, 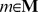, all possible state 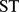, and a revocation list 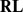, if 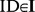 is not revoked before or at time 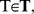 then for 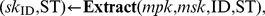 
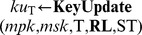
, and 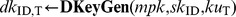, the following equation holds.



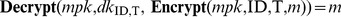



The property of indistinguishability under adaptively chosen identity and chosen plaintext attack (IND-ID-CPA) is considered a basic requirement for provably secure IBE schemes. For RIBE scheme, we define indistinguishability under adaptively chosen revocable identity and chosen plaintext attack (IND-RID-CPA) by the following game between an adversary and a challenger. Note that the security model captures realistic threats including decryption key exposure [13].

### 

#### Definition 6

*Let* 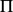 *be a RIBE scheme, we say that* 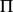 *is IND-RID-CPA secure if any PPT adversary* 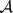 *has negligible advantage in this following experiment:*



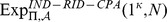


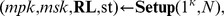


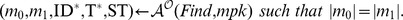


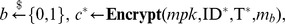


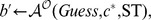


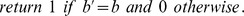



*The adversary* 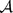 *'s advantage is defined as follows.*



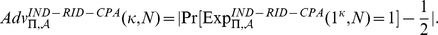



In the above experiment, 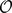 is a set of oracles defined as follows.

- **Extract Oracle**: For 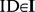, it runs 
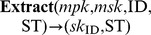
, then returns 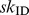 and update state 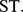

- **KeyUpdate Oracle**: For 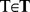, it runs 
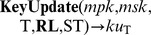
, then returns 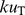.
- **Revoke Oracle**: For 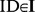 and 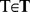, it runs 
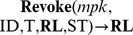
, then returns the updated revocation list 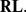

- **DKeyGen Oracle**: For 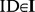 and 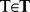, it runs 
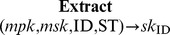
 and 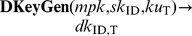, then returns 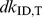.

The adversary 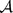 is allowed to query above oracles with the following restrictions:

- **KeyUpdate Oracle** and **Revoke Oracle** can be queried on time which is greater than or equal to the time of all previous queries, i.e. the adversary is allowed to query only in non-decreasing order of time.
- **Revoke Oracle** cannot be queried on time 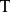 if **KeyUpdate Oracle** was queried on 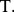

- If 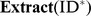 was queried, then 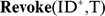 must be queried for 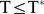.
- **DKeyGen Oracle** cannot be queried on time 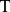 before **KeyUpdate Oracle** was queried on 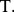

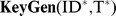
 cannot be queried.

This definition naturally extends to the chosen ciphertext scenario where the adversary is further granted access to a **Decrypt Oracle** that, on input of a ciphertext 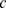 and a pair 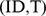, it returns 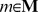 or 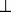 by running 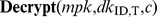. Of course, **Decrypt Oracle** cannot be queried on the ciphertext 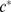 for the pair 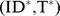.

## Our Construction

In this section, we propose an efficient and provable secure RIBE scheme by exploiting Lewko and Waters IBE scheme [9] and 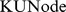 algorithm.

**Setup** The PKG runs composite order bilinear group generator 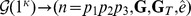, chooses 
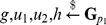
 and 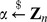. The PKG publishes the public system parameters as follows.



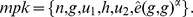



The master secret keys are 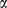 and a generator of 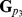.

**Extract** The PKG chooses an unassigned leaf 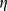 from 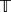 at random, and stores 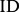 in the node 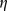. For each node 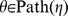, PKG performs as follows.

- Recall 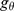 if it was defined. Otherwise, 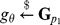 and store 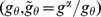 in the node 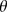.
- Choose 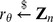 and 
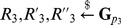
 at random. Note that we can get a random elements of 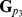 by taking a generator of 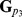 and raising it to random exponents modulo 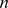.
- Compute 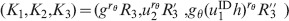.
- Return 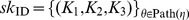.

**KeyUpdate** The PKG parses 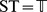, and performs the following steps for each node 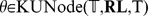.

- Retrieve 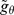 (note that 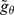 is always pre-defined in the 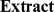 algorithm).
- Choose 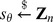 and 
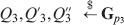
.
- Compute 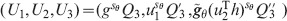.
- Return 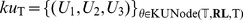.

**DKeyGen** User parses 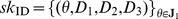 and 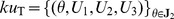. If 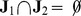, then outputs error symbol 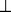. Otherwise, user chooses 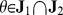 and 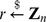 and outputs



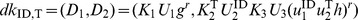



**Encrypt** A sender chooses a random integer 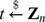 and outputs



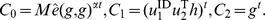



**Decrypt** The receiver parses 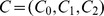 and 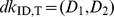 and outputs



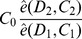



**Revoke** Let 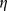 be the leaf node associated with 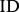. The PKG updates the revocation list by 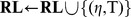 and returns the updated revocation list.

The correctness of our RIBE construction can be verified as follow.



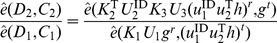


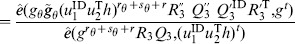


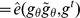


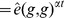



## Security Proofs

To prove the security of our RIBE scheme, we first define three additional structures: semi-functional ciphertexts, semi-functional private keys and semi-functional update keys. For the semi-functional type, we let 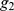 denote a fixed generator of the subgroup 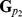.

- **Semi-functional Ciphertext**: A normal ciphertext 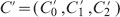 is first generated by the encryption algorithm. It then chooses 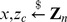 and sets:
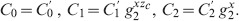

  The semi-functional ciphertext is 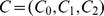.

- **Semi-functional Private Key**: A normal private key 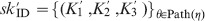 is generated by the private key generation algorithm for an identity 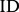. It then chooses 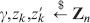 and sets:
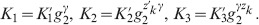

  The semi-functional private key is 
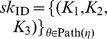
.

- **Semi-functional Update Key**: A normal update key 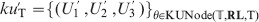 is generated by the update key generation algorithm. It then chooses 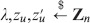 and sets:
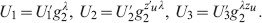

  The semi-functional update key is 
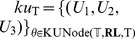
.

- **Semi-functional Decryption Key**: A normal decryption key 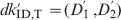 is generated by the decryption key generation algorithm. It then chooses 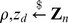 and sets:
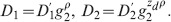

  The semi-functional decryption key is 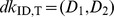.

Note that when a semi-functional decryption key is used to decrypt a semi-functional ciphertext, the decryption algorithm will compute the blinding factor multiplied by the additional term 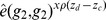. If 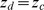, decryption will still work. In this case, the decryption key is nominally semi-functional. In our proof, normal decryption keys are generated by normal subkeys of private keys and normal subkeys of update keys, while semi-functional decryption keys are generated by semi-functional subkeys of private keys and normal subkeys of update key, or normal subkeys of private keys and semi-functional subkeys of update keys.

There are two types of adversaries in simulation. Type-I adversary issues private key queries on the challenge identity 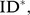 but the challenge identity should be revoked before the challenge time 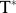; Type-II adversary will never issue private key queries on the challenge identity. Obviously, if a RIBE scheme is secure against Type-I adversary, it is definitely secure against Type-II adversary. For this reason, we only consider Type-I adversary in the following security proofs.

Denote by 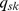 and 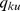 the number of private key queries for non-challenge identities and update key queries for non-challenge time issued by an adversary, respectively. Denote by 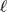 the maximum node number a private key involves, and those nodes are not on the path from the root node to the challenge node 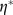.

We give our proof as a sequence of games, which are defined in the order as follows.

- Game_A_: The actual RIBE security game, where all private keys, update keys, decryption keys and the challenge ciphertext are normal.
- Game_R_: The restricted game, is the actual security game except that adversary can not issue private key queries for 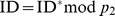 and update key queries for 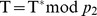. Note that adversary can issue private key queries for 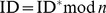, but 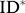 should be revoked before 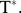

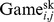
: The restricted security game where the challenge ciphertext, all 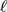 subkeys of first 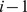 private keys and all first 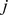 subkeys 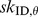 of the 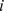-th private key 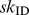 are semi-functional, while all subkeys of the rest private keys and all subkeys of update keys are normal. Here 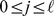, 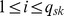 and 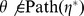.

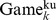
: The restricted security game where the challenge ciphertext, all 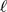 subkeys of all private keys, and subkeys 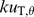 of the first 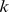 update key 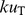 are semi-functional, while the rest subkeys of 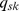 private key and the rest subkeys of 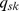 update keys are normal. Here 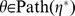. It is obvious that 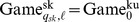.
- Game_F_: The final game, is the same as security game 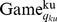 except that the challenge ciphertext is a semi-functional encryption of a random message.

Next, we prove the indistinguishability of those games by following lemmas.

### 

#### Lemma 1

*Suppose there exists an algorithm* 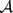 *such that* 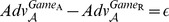 *, then we can build an algorithm* 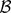 *with advantage* 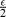 *in breaking Assumption 2.*

*Proof*. Given 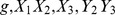, algorithm 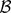 can simulate 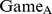 with 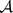. Assume that 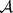 produces identities 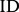 and 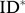 such that 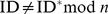 and 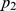 divides 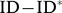 with probability 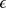 (If 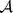 fails to do this, 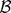 simply guesses at random). 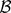 uses these identities to produce a nontrivial factor of 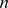 by computing 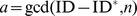. Set 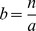, and consider the following three cases:

- Case 1 one of 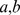 is 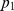, and the other is 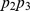

- Case 2 one of 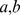 is 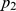, and the other is 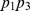

- Case 3 one of 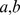 is 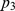, and the other is 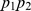

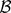
 can determine if Case 1 has occurred by testing if either of 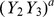 or 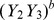 is the identity element. If this happens, we will suppose that 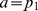 and 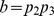 without loss of generality. 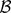 can then learn whether 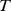 has a 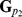 component or not by testing if 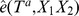 is the identity element. If it is not, then 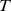 has a 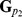 component.


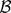
 can determine if Case 2 has occurred by testing if either of 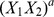 or 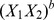 is the identity element. Assuming that 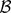 has already ruled out Case 1 and neither of them is the identity element, then Case 2 has occurred. 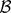 can learn which of 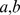 is equal to 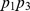 by testing which of 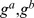 is the identity. Without loss of generality, we assume that 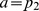 and 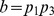. Then, 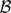 can learn whether 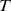 has a 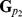 component or not by testing if 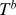 is the identity element. If it is not, then 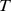 has a 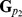 component.


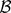
 can determine that Case 3 has occurred when the tests for both Cases 1 and Case 2 fail. It can learn which of 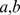 is equal to 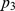 by testing which of 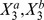 is the identity. Without loss of generality, we assume that 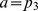. 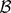 can learn whether 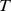 has a 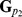 component or not by testing whether 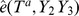 is the identity. If it is not, then 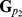 has a 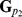 component.

This completes the proof.

□

#### Lemma 2

*Suppose there exists an algorithm* 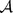 *such that* 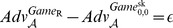 *, then we can build an algorithm* 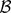 *with advantage* 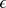 *in breaking Assumption 1.*

*Proof*. 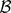 first receives 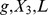, then simulates 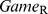 or 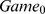 with 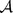. 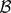 chooses 
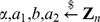
, sets public parameters as 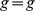, 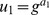, 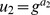, 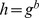, and sends the public parameters to 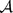.

- When 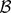 is asked to provide a update key with time period 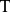. For each node 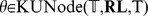, 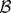 performs the following steps.
  1. Retrieve 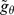 (Note that 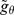 is always pre-defined in the 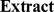 algorithm).
  2. Choose 
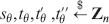
.
  3. Compute 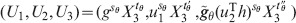.
  4. Return 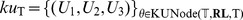.
- When 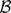 is asked for a private key with identity 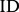. For each node 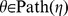 where 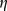 is the leaf node assigned to 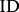, 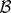 performs the following steps.
  1. Recall 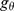 if it was defined. Otherwise, 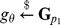 (We can do this by by taking the generator of 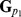, 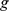, and raising it to random exponents modulo 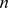) and store 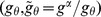 in the node 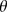.
  2. Choose 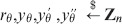.
  3. Compute 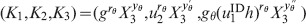.
  4. Return 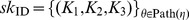.
- When 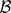 is asked for a decryption key with identity 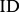 and time period 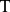, then 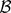 successively runs the 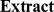 algorithm, 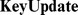 algorithm and 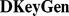 algorithm.

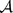
 sends 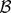 two message, 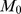 and 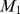, and a challenge identity, 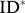, challenge time period, 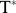. 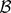 chooses 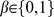 randomly. The ciphertext is formed as follows.



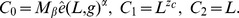



This implicitly sets 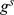 equal to the 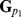 part of 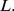 If 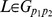, then this is a semi-functional ciphertext with 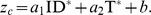 We note that the value of 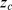 modulo 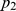 is not correlated with the values of 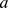 and 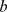 modulo 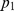, so 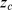 is properly distributed. If 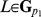, this is a normal ciphertext. Hence, simulator 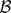 can use the output of 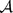 to distinguish between these possibilities for 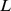.

This completes the proof.

□

#### Lemma 3

*Suppose there exists an algorithm* 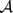 *such that* 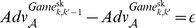 *, then we can build an algorithm* 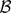 *with advantage* 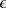 *in breaking Assumption 2.*

*Proof*. 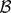 first receives 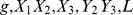, and picks 
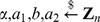
, then 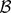 sets the public parameters as 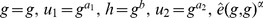 and sends the public parameters to 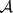.

- When 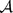 issues the 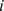-th private key query for all subkeys corresponding to the challenge identity, or subkeys that associated with nodes are not on the path from the node associated with challenge identity to the root node, 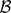 generates normal private keys by calling the normal private key generation algorithm. Otherwise, 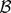 generate the *j*-th subkey, associated with those 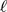 subkeys, of the *i*-th private key as follows.

1. For 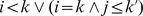 and 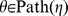

  - Recall 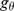 if it was defined. Otherwise, 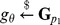 and store 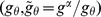 in the node 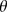.
  - Choose 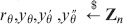 randomly.
  - Compute 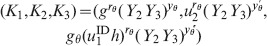.
  - Return 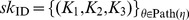.
2. For 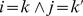 and 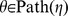,
  - Recall 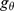 if it was defined. Otherwise, 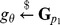 and store 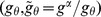 in the node 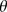.
  - Choose 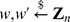 randomly.
  - Compute 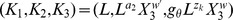.
  - Return 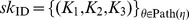.
3. For 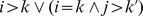, 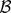 generates normal private keys by calling the normal private key generation algorithm.

- When 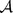 issues a update key query with time period 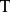, then 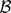 generates normal update keys by calling the normal update key generation algorithm.
- When 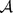 issues a a decryption key query with identity 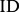 and time period 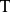, then 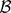 successively runs the 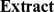 algorithm, 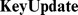 algorithm, and 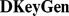 algorithm.

At some point 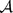 sends two messages, 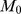 and 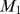, a challenge identity 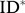, and a challenge time period 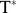 to 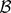. 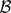 sets 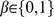 randomly. The challenge ciphertext is formed as follows.
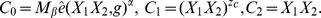



We note that this sets 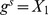 and 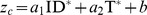. Since 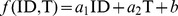 is a pairwise independent function modulo 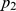, as long as 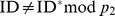 and 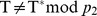, 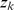 and 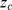 will seem randomly distributed to 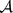.

If 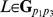, then 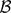 has properly simulated Game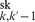. If 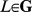, then 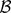 has properly simulated Game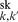. Hence, 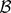 can use the output of 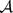 to distinguish between these possibilities for 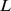.

This completes the proof.

□

#### Lemma 4

*Suppose there exists an algorithm* 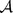 *such that* 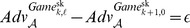 *, then we can build an algorithm* 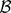 *with advantage* 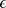 *in breaking Assumption 2.*

*Proof.* This proof is analogous to the proof of lemma 3.

□

#### Lemma 5

*Suppose there exists an algorithm* 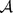 *such that* 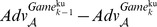 *, then we can build an algorithm* 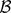 *with advantage* 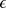 *in breaking Assumption 2.*

*Proof.* 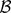 first receives 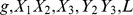, and picks 
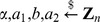
, then 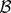 sets the public parameters as 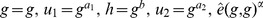 and sends the public parameters to 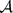.

- When 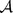 issues private key query for the challenge identity or subkeys that associated with nodes are not on the path from the node associated with challenge identity to the root node, 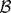 generates normal private keys by calling the normal private key generation algorithm. Otherwise, for each node 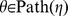, 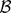 performs as follows.
  1. Recall 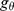 if it was defined. Otherwise, 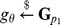 and store 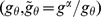 in the node 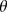.
  2. Choose 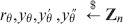 randomly.
  3. Compute 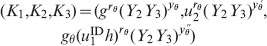.
  4. Return 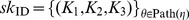.
- When 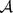 issues the update key query for the challenge time period, 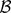 generates normal update keys by calling the normal update key generation algorithm. Otherwise, 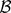 performs as follows.

1. For 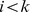. When 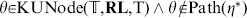, 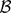 calls the normal update key generation algorithm. When 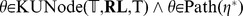, 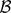 acts as follow. Note that there is only one node that satisfies this condition in each update node set.
  - Retrieve 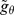.
  - Choose 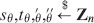.
  - Compute 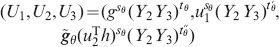.
  - Return 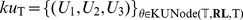.
2. For 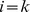. When 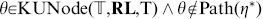, 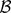 generates normal update keys by calling the normal update key generation algorithm. When 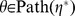, 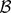 performs as follows.
  - Retrieve 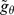.
  - Choose 
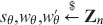
.
  - Compute 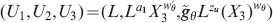.
  - Return 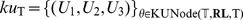.
  - Here we note that 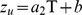 and 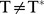, therefore both 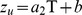 and 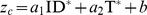 seem random in adversary's view. If 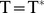, namely we transform update key with time period 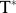, then we can not ensure that 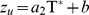 and 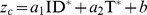 seem random in adversary's view.
3. For 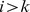. When 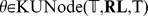, 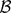 generates normal update keys by calling the normal update key generation algorithm.

- When 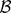 is asked for a decryption key with identity 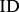 and time period 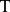, 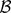 successively runs the 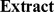 algorithm, 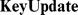 algorithm and 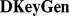 algorithm.

At some point 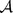 sends two messages, 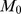 and 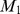, a challenge identity 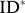, and a challenge time period 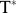 to 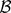. 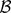 sets 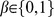 randomly. The challenge ciphertext is formed as follows.



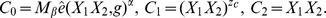



If 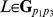, then 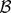 has properly simulated 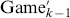. If 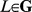, then 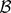 has properly simulated 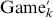. Hence, 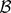 can use the output of 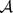 to distinguish between these possibilities for 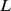.

This completes the proof.

□

#### Lemma 6

*Suppose there exists an algorithm* 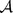 *such that* 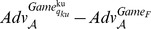 *, then we can build an algorithm* 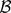 *with advantage* 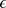 *in breaking Assumption 3.*

*Proof.* 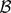 first receives 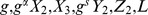, chooses 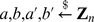, then 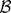 sets the public parameters as 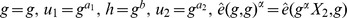 and sends the public parameters to 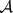.

- When 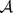 issues private key queries with the challenge identity or nodes on the path from challenge identity node to root node, 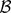 generates normal private keys by calling the normal private key generation algorithm. Otherwise, for each node 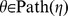, 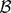 performs as follows.
  1. Recall 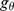 if it was defined. Otherwise, 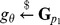 and store 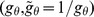 in the node 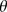.
  2. Choose 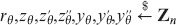 randomly.
  3. Compute 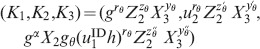.
  4. Return 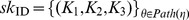.
- When 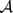 issues update key query with the challenge time period, 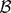 generates normal update keys by calling the normal update key generation algorithm. Otherwise, 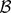 performs as follows.
  1. If 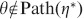, 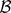 generates normal update keys by calling the normal update key generation algorithm.
  2. If 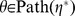, 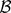 performs the following steps. Note that there is only one such node in each time period 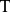.
    1. Retrieve 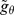 (note that 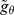 is always pre-defined in the 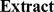 algorithm).
    2. Choose 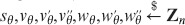.
    3. Compute 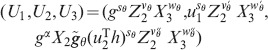.
    4. Return 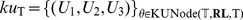.
- When 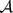 issues decryption key query with identity 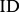 and time period 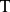, 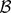 successively runs the 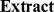 algorithm, 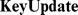 algorithm and 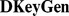 algorithm.

At some point 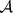 sends two messages, 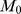 and 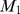, a challenge identity 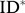, and a challenge time period 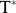 to 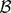. 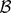 sets 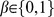 randomly. The challenge ciphertext is formed as follows.



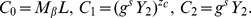



Here 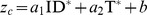. We note that the value of 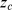 only matters modulo 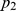, whereas 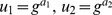 and 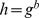 are elements of 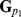, so when 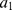, 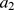 and 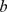 modulo 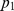 are chosen randomly modulo 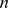, there is no correlation between the values of 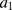, 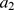 and 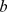 modulo 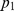 and the value 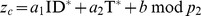.

If 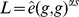, then this is a properly distributed semi-functional ciphertext with message 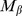. If 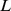 is a random element of 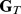, then this is a semi-functional ciphertext with a random message. Hence, 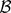 can use the output of 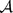 to distinguish between these possibilities for 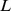.

This completes the proof.

□

#### Theroem 1

*If above lemmas hold, then our RIBE scheme is adaptively secure under assumption 1, 2 and 3. More precisely, for any adversary* 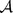 *that makes at most* 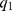 *private key queries,* 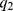 *update key queries against our RIBE scheme, we have*



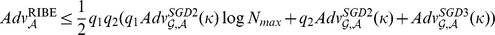



*Proof.* If above assumptions hold, then we have shown by the previous lemmas that the real security game is indistinguishable from 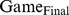, in which the value of 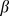 is information-theoretically hidden from the adversary. Hence the adversary can attain no advantage in breaking our RIBE scheme.

This completes the proof.

## Conclusion

In this paper, we presented a scalable RIBE scheme with decryption key exposure resilience in the composite order group setting by combining Lewko and Waters' IBE scheme and complete subtree method, and proved our proposed RIBE scheme to be adaptive-ID secure by employing the recent dual system encryption methodology. Compared to existing adaptive-ID secure LV-RIBE scheme and SE-RIBE scheme, our proposed RIBE construction is more efficient in term of ciphertext size, public parameters size and decryption cost at price of a little looser security reduction. In our future work, we will focus on constructing an adaptive-ID secure RIBE scheme with decryption key exposure resilience in the prime order group setting and devising an adaptive-ID secure RIBE scheme that can resist decryption key exposure attack with a tighter reduction.

## References

[1] Shamir A (1985) Identity-based cryptosystems and signature schemes. In: Advances in Cryptology- CRYPTO 84. California, USA: Springer Berlin Heidelberg, volume 196 of Lecture Notes in Computer Science, pp. 47–53.

[2] Boneh D, Franklin M (2001) Identity-based encryption from the weil pairing. In: Advances in Cryptology - CRYPTO 2001. California, USA: Springer Berlin Heidelberg, volume 2139 of Lecture Notes in Computer Science, pp. 213–229.

[3] Canetti R, Goldreich O, Halevi S (2004) The random oracle methodology, revisited. Journal of the ACM 51: 557–594.

[4] Canetti R, Halevi S, Katz J (2004) Chosen-ciphertext security from identity-based encryption. In: Advances in Cryptology - EUROCRYPT 2004. Interlaken, Switzerland: Springer Berlin Heidelberg, volume 3027 of Lecture Notes in Computer Science, pp. 207–222.

[5] Boneh D, Boyen X (2004) Efficient selective-id secure identity-based encryption without random oracles. In: Advances in Cryptology - EUROCRYPT 2004. Interlaken, Switzerland: Springer Berlin Heidelberg, volume 3027 of Lecture Notes in Computer Science, pp. 223–238.

[6] Waters B (2005) Efficient identity-based encryption without random oracles. In: Advances in Cryptology - EUROCRYPT 2005. Aarhus, Denmark: Springer Berlin Heidelberg, volume 3494 of Lecture Notes in Computer Science, pp. 114–127.

[7] Gentry C (2006) Practical identity-based encryption without random oracles. In: Advances in Cryptology - EUROCRYPT 2006. St. Petersburg, Russia: Springer Berlin Heidelberg, volume 4004 of Lecture Notes in Computer Science, pp. 445–464.

[8] Waters B (2009) Dual system encryption: Realizing fully secure ibe and hibe under simple assumptions. In: Advances in Cryptology - CRYPTO 2009. California, USA: Springer Berlin Heidelberg, volume 5677 of Lecture Notes in Computer Science, pp. 619–636.

[9] Lewko A, Waters B (2010) New techniques for dual system encryption and fully secure hibe with short ciphertexts. In: Theory of Cryptography - TCC 2010. Zurich, Switzerland: Springer Berlin Heidelberg, volume 5978 of Lecture Notes in Computer Science, pp. 455–479.

[10] Zhang Y, Wang L, Zhang Y, Li X (2012) Toward a temporal network analysis of interactive wifiusers. Europhysics Letters 98: 68002.

[11] Boldyreva A, Goyal V, Kumar V (2008) Identity-based encryption with efficient revocation. In: Proceedings of the 15th ACM Conference on Computer and Communications Security. Virginia, USA: ACM, CCS 2008, pp. 417–426.

[12] Libert B, Vergnaud D (2009) Adaptive-id secure revocable identity-based encryption. In: Topics in Cryptology - CT-RSA 2009. California, USA: Springer Berlin Heidelberg, volume 5473 of Lecture Notes in Computer Science, pp. 1–15.

[13] Seo J, Emura K (2013) Revocable identity-based encryption revisited: Security model and construction. In: Public Key Cryptography - PKC 2013. Nara, Japan: Springer Berlin Heidelberg, volume 7778 of Lecture Notes in Computer Science, pp. 2161–234.

[14] Park S, Lee K, Lee D (2013). New constructions of revocable identity-based encryption from multilinear maps. Cryptology ePrint Archive. Report 2013/880.

[15] Lee K, Lee D, Park J (2014). Efficient revocable identity-based encryption via subset difference methods. Cryptology ePrint Archive. Report 2014/132.

[16] Sahai A, Waters B (2005) Fuzzy identity based encryption. In: Advances in Cryptology - EUROCRYPT 2005. Aarhus, Denmark: Springer Berlin Heidelberg, volume 3494 of Lecture Notes in Computer Science, pp. 457–473.

[17] Naor D, Naor M, Lotspiech J (2001) Revocation and tracing schemes for stateless receivers. In: Advances in Cryptology - CRYPTO 2001. California, USA: Springer Berlin Heidelberg, volume 2139 of Lecture Notes in Computer Science, pp. 41–62.

[18] Libert B, Vergnaud D (2009) Towards black-box accountable authority ibe with short ciphertexts and private keys. In: Public Key Cryptography - PKC 2009. California, USA: Springer Berlin Heidelberg, volume 5443 of Lecture Notes in Computer Science, pp. 235–255.

